# Confirmatory factor analysis of the Evidence-Based Practice Attitudes Scale with school-based behavioral health consultants

**DOI:** 10.1186/s13012-018-0804-z

**Published:** 2018-08-22

**Authors:** Clayton R. Cook, Chayna Davis, Eric C. Brown, Jill Locke, Mark G. Ehrhart, Gregory A. Aarons, Madeline Larson, Aaron R. Lyon

**Affiliations:** 1 University of Minnesota, 341 Education Sciences Bldg, 56 East River Road, Minneapolis, MN 55455 USA; 2 University of Washington, 6200 NE 74th Street, Suite 100, Seattle, WA 98115 USA; 3 Department of Public Health Sciences, Miller School of Medicine, University of Miami, 1120 NW 14th Street, Office 104, Miami, FL 33136 USA; 4 University of Central Florida, 4111 Pictor Lane, Orlando, FL 32816-1390 USA; 5 University of California San Diego, 9500 Gilman Drive (0812), La Jolla, CA 92093 USA; 6 Child and Adolescent Services Research Center, La Jolla, USA

**Keywords:** Evidence-based practice, Attitudes, Education sector, Reliability, Structural validity, Confirmatory factor analysis, Evidence-Based Practice Attitudes Scale, Implementation

## Abstract

**Background:**

The Evidence-Based Practice Attitude Scale (EBPAS) is a widely used tool, but it has not been adapted and validated for use in schools, the most common setting where youth access behavioral health services. This study examined the factor structure, psychometric properties, and criterion-related validity of the school-adapted EBPAS in a sample of school-based behavioral health consultants.

**Method:**

A research team comprised of experts in implementation of evidence-based practices in schools along with the original developer adapted the EBPAS for the school setting. The adapted instrument was administered to a representative sample (*n* = 196) of school-based behavioral health consultants to assess the reliability and structural validity via a series of confirmatory factor analyses.

**Results:**

The original EBPAS factor structure was confirmed, with the final model supporting four first-order factors that load onto a second-order factor capturing general attitudes toward evidence-based practice. Correlations among the subscales indicated both unique and shared variance. Correlations between EBPAS scores and consultant variables demonstrated differential criterion-related validity, with the total score and the Requirements and Openness subscales demonstrating the strongest correlations.

**Conclusions:**

The adapted EBPAS performed well when administered to behavioral health consultants operating in the educator sector, supporting the relevance of assessing attitudes in school settings. Potential directions for future research and applications of the EBPAS in schools and other service sectors are discussed.

**Electronic supplementary material:**

The online version of this article (10.1186/s13012-018-0804-z) contains supplementary material, which is available to authorized users.

Research has identified numerous determinants that either enable or obstruct the successful implementation of evidence-based practices (EBP), including outer context, inner context, and innovation-specific factors [1–3]. Notwithstanding the influence of these factors, successful implementation rests with the decisions and behaviors of those who are most closely connected to the adoption and delivery of EBP, such as designated providers and embedded consultants within the service setting. Indeed, mounting evidence suggests that individual-level factors play a central role in predicting implementation outcomes [4, 5]. One individual-level factor, attitudes toward evidence-based practice, has garnered significant attention across service sectors as an important determinant that is linked to successful implementation [6–8].

## Evidence-Based Practice Attitude Scale

According to Crano and Prislin [9], attitudes reflect evaluative judgments based on the integration of specific behavioral beliefs that impact a person’s motivation, ambivalence, and resistance to perform a specific action. When focused specifically on the implementation of EBP, those attitudes reflect a person’s favorable or unfavorable evaluative judgments about the adoption and delivery of EBP. Aarons [6] was the first to design a measure dedicated to capturing attitudes related to the implementation of EBP. Through work in community-based mental health settings, the Evidence-Based Practice Attitudes Scale (EBPAS) was developed to include four subscales that capture distinct yet interrelated constructs: (1) willingness to adopt EBPs given their *intuitive appeal*; (2) willingness to adopt new practices *if required*; (3) general *openness* toward new or innovative practices; and (4) perceived *divergence* of usual practice with academically developed or research-based practices [4]. Since the original study [4], the EBPAS has been used extensively in research across different implementation contexts and providers [10–12]. One of the most comprehensive validation studies to date involved administering the EBPAS to over 1000 mental health providers from 100 different community-based organizations across 26 states in the USA [8]. Results supported the scale’s second-order factor structure (i.e., four subscales) and demonstrated adequate reliability of the subscales and total scale.

## Gaps in EBPAS research

There is a need for studies that can determine the extent to which existing implementation findings and products, such as the EBPAS, are reliable and valid in novel contexts. Cross validation in other settings is key to advance the multi-disciplinary field of implementation science by determining whether specific constructs and instruments are context-dependent or context-independent. Despite the existing research on the EBPAS and its potential to inform both implementation science and practice, psychometric findings regarding the internal factor structure and criterion validity have not been replicated in schools.

The educational sector offers unique opportunities to promote youth behavioral health given that over 70% of youth who receive behavioral health services in the USA do so in schools [13, 14]. Behavioral health services delivered in schools often are not evidence-based nor delivered with sufficient fidelity, resulting in a significant waste of resources (e.g., funds invested in the research) and a missed opportunity to promote public health outcomes [15–17]. Most schools have a diverse set of personnel who can support the delivery of behavioral health services [16]. Among them are school-based behavioral health consultants who frequently operate as implementation intermediaries that are tasked with supporting the delivery of EBP across multiple levels of care ranging from prevention to treatment [18]. Because their intermediary role positions them as gatekeepers during behavioral health implementation efforts, the attitudes of these personnel may be critical to EBP implementation success.

The research on the EBPAS has focused exclusively on providers, albeit across multiple contexts [10–12], with limited to no research examining whether the construct validity of the measure holds for other implementation stakeholders, such as consultants or intermediaries. Further, it is likely that a consultant’s attitudes toward evidence-based practice would be associated with consultant-relevant variables linked to provider-level implementation outcomes. However, research offers no empirical guidance on whether a consultant’s attitudes predict other variables relevant to implementation, such as a consultant’s embeddedness (i.e., activity, visibility, and collaboration) [19, 20], use of implementation strategies to promote EBP adoption and delivery [21, 22], and consultant self-efficacy (i.e., belief in one’s ability to promote provider behavior change) with regard to promoting provider behavior change [23].

## Purpose of the present study

The purpose of this paper was to extend the research on the EBPAS by examining the construct validity of an adapted version of the EBPAS through a series of confirmatory factor analyses with a sample of school-based behavioral health consultants. A secondary aim was to examine whether the school-adapted EBPAS predicted three variables related to behavioral health consultants’ EBP implementation activities, including their embeddedness, use of implementation strategies, and self-efficacy.

## Method

### Sample

The study sample included members of a statewide educational and behavioral health organization on the West Coast of the USA committed to the delivery of EBPs for students who exhibit mental and behavioral health concerns. The majority of organization members were in positions that support the delivery of behavioral health EBPs. Of the survey responses received, 196 participants (89%) completed at least 80% of questions in the section pertaining to consultation and were thus included in analyses. Complete demographic information for participants is shown in Table 1.

**Table 1:** Demographics of survey respondents (*n* = 196)

Characteristic	*n*	%
Gender		
Male	39	19.9
Female	155	79.1
Prefer not to disclose/missing	2	1.0
Ethnicity		
American Indian or Alaska Native	2	1.0
Asian	8	4.1
Black or African American	10	5.1
Hispanic or Latino	17	8.7
Multirace	12	6.1
Other	3	1.5
White/Non-Hispanic	137	69.9
Prefer not to disclose/missing	7	3.6
Highest degree earned		
BA/BS	1	0.5
Master’s degree	175	89.3
Doctoral degree (PhD, EdD, PsyD)	16	8.2
Other, not specified	1	0.5
Prefer not to disclose/missing	3	1.5
Years of experience		
0 to 3 years	3	1.5
3 to 5 years	9	4.6
6 to 10 years	47	24.0
11 to 20 years	94	48.0
> 20 years	40	20.4
Prefer not to disclose/missing	3	1.5

*n*, sample size

### Procedures

This study was reviewed and determined to be exempt by the Human Subjects Institutional Review Board. Approval was obtained from the participating statewide organizational leadership. Data were collected via an online survey, distributed through a series of emails to organization members. Prior to constructing and administering the survey, school-based implementation experts adapted EBPAS items for the educational context in collaboration with the developer of the original measure. Adaptations consisted of changing item wording to ensure construct equivalence for the target respondents (i.e., school-based practitioners), while preserving the integrity of the original items/constructs to ensure appropriateness to the school context [24]. Thus, all items were maintained with changes only made to item wording, such as replacing the word “supervisor” with “school administrator,” “clinician” with “school personnel,” and “agency” with “school.” One additional item was included to the EBPAS to capture whether the respondent would adopt an EBP if it was required by the school district.

In the fall, members were sent an e-mail asking and recruiting them to participate in an online survey study. The current study was part of a larger project examining school-based behavioral health consultants’ perceptions of the implementation of school-based EBPs and employed best practices in designing a web-based survey (e.g., visual ease, clear instructions, sending the survey, reminders) [25]. For this analysis, only items from the EBPAS and criterion-related ratings of implementation strategies, embeddedness, and self-efficacy were included.

### Measures

#### Evidence-Based Practice Attitudes Scale (EBPAS)

The original EBPAS was developed to assess the degree to which providers possess favorable attitudes toward the adoption and delivery of EBPs [10–12]. The original scale includes a total of 15 items capturing four subscales: Appeal, Requirements, Openness, and Divergence. The Requirements subscale includes three items while the other three subscales include four items each. An additional item was included under the Requirements subscale to capture attitudes toward EBP if “required by school district.” The Divergence scale was reversed scored so higher scores corresponded to more favorable attitudes like the other scales. Respondents rated each item on a 5-point scale ranging from *Not at all* to a *Very Great Extent*. The EBPAS has demonstrated adequate internal consistency reliability as well as convergent and discriminant validity from related scales [8].

#### Criterion-related variables

Items assessing three criterion-related variables relevant to consultation were included: consultant embeddedness, use of implementation strategies, and consultant self-efficacy.

##### Consultant embeddedness

Items were adapted from the Expanded School Mental Health Collaboration Instrument [19] to capture consultant embeddedness (i.e., degree of visibility, presence, and collaboration) in a given school. In particular, 13 items from the Outreach and Approach subscale capturing clinician embeddedness were adapted. Scale items are rated on a 4-point scale (*Strongly Disagree* to *Strongly Agree*) and summed to create a total score. The Outreach and Approach scale has demonstrated acceptable reliability and validity [19]. In this study, the scale also demonstrated acceptable internal consistency (*α* = .89).

##### Use of implementation strategies

Fifteen items assessing whether respondents used a subset of implementation strategies selected from an existing compilation that were relevant to the consultant role [26] were included as a self-report measure of consultant implementation-oriented behavior (see Additional file 1 for list of strategies). Respondents rated *Yes* or *No* regarding their use of each of the 15 strategies, with the DV serving as the total number of techniques used to support provider implementation.

##### Consultant self-efficacy

Four items drawn from the Generalized Self-Efficacy scale [27] were adapted and included in the survey to assess consultant self-efficacy, which assessed beliefs to produce desired effects when supporting teachers to adopt and deliver EBPs. Example items included “I am able to increase the fidelity with which a teacher implements the intended intervention as planned” and “I feel confident in ensuring that the intervention is appropriate and fits well with teachers’ classroom environment.” The items were rated on a 5-point scale ranging from *Not at All* to *Very Great Extent*. In this study, the scale demonstrated acceptable internal consistency (*α* = .82).

### Data analytic approach

The data analytic procedure involved examining the construct validity of the school-adapted EBPAS via a series of confirmatory factor analyses (CFA) using weighted least squares means and variances (WLSMV) estimation with delta parameterization for the ordered-categorical scale items, as employed in M*plus* [28]. The fit of each model was determined across several indices (e.g., chi-square statistic, comparative fit index [CFI], the Tucker-Lewis index [TLI], root mean square error of approximation [RMSEA]) with values of the CFI and TLI greater than .95 and values of the RMSEA less than or equal to .05 as indicative of good model fit to the data [29–33]. Standardized factor loadings (*ß*) less than .55 were deemed poorly performing items that required further examination. The measurement model from the original EBPAS was tested first, followed by subsequent modifications based on resulting model modification indices and theoretical justification. Finally, evidence supporting the construct validity was examined via correlational analyses testing associations between EBPAS scores and criterion-related variables.

## Results

### Summary statistics

Summary statistics for the EBPAS scale and subscale items in the form of means, standard deviations, and estimates of subscale internal consistency (coefficient alphas) are depicted in Table 2. Descriptive statistics indicated that the Openness subscale had the highest mean and smallest standard deviation, while the Requirements subscale had the lowest mean and most dispersion. Statistics and graphed data of the response distributions for each of the measures and subscales were examined to assess skewness, kurtosis, and normality. Inspection of these data indicated that all subscales had relatively normally distributed data, with slight negative skewness for Requirements, Openness, and Appeal subscales, and slight positive skewness for the Divergence subscale. With regard to reliability, the subscales showed strong internal consistency (i.e., *α* > .80), with the exception of the Divergence subscale (*α* = .63).

**Table 2:** Summary statistics for the four EBPAS subscales

EBPAS subscales	*n*, *M*, ± SD	*α*	*Ω*
Requirements: Perceptions regarding if delivering EBPs is required	185, 3.07 ± .87	.96	.97
It was required by your supervisor/administrator?	3.03 ± 0.88		
It was required by your school?	3.06 ± 0.87		
It was required by your district?	3.08 ± 0.88		
It was required by your state?	3.12 ± 0.85		
Appeal: Perceptions regarding if delivering EBPs is found to be appealing	182, 3.20 ± .77	.83	.90
It was intuitively appealing?	2.90 ± 0.92		
It “made sense” to you?	3.20 ± 0.75		
It was being used by colleagues who were happy with it?	3.23 ± 0.80		
You felt you had enough training to use it correctly?	3.46 ± 0.68		
Openness: Perceptions regarding openness to delivering EBPs	183, 3.24 ± .78	.82	.87
I like to use new types of methods/interventions to help students.	3.19 ± 0.78		
I am willing to try new types of methods/interventions even if I have to follow a teaching/training manual.	3.30 ± 0.78		
I am willing to use new and different types of methods/interventions developed by researchers.	3.34 ± 0.69		
I would try new methods/interventions even if it were very different from what I am used to doing.	3.11 ± 0.89		
Divergence: Perceptions that diverge from delivering EBPs	159, 3.27 ± .84	.63	.67
I know better than academic researchers how to care for students.	3.05 ± 0.98		
Research-based teaching methods/interventions are not useful in practice.	3.39 ± 0.72		
Professional experience is more important than using manualized methods/interventions.	2.72 ± 0.98		
I would not use manualized methods/interventions.	3.63 ± 0.73		
Total score	159, 12.78 ± 2.17		

*n* sample size, *M* mean score, *SD* standard deviation, *α* alpha, *Ώ* omega

### Confirmation factor analyses

**Fig. 1:**
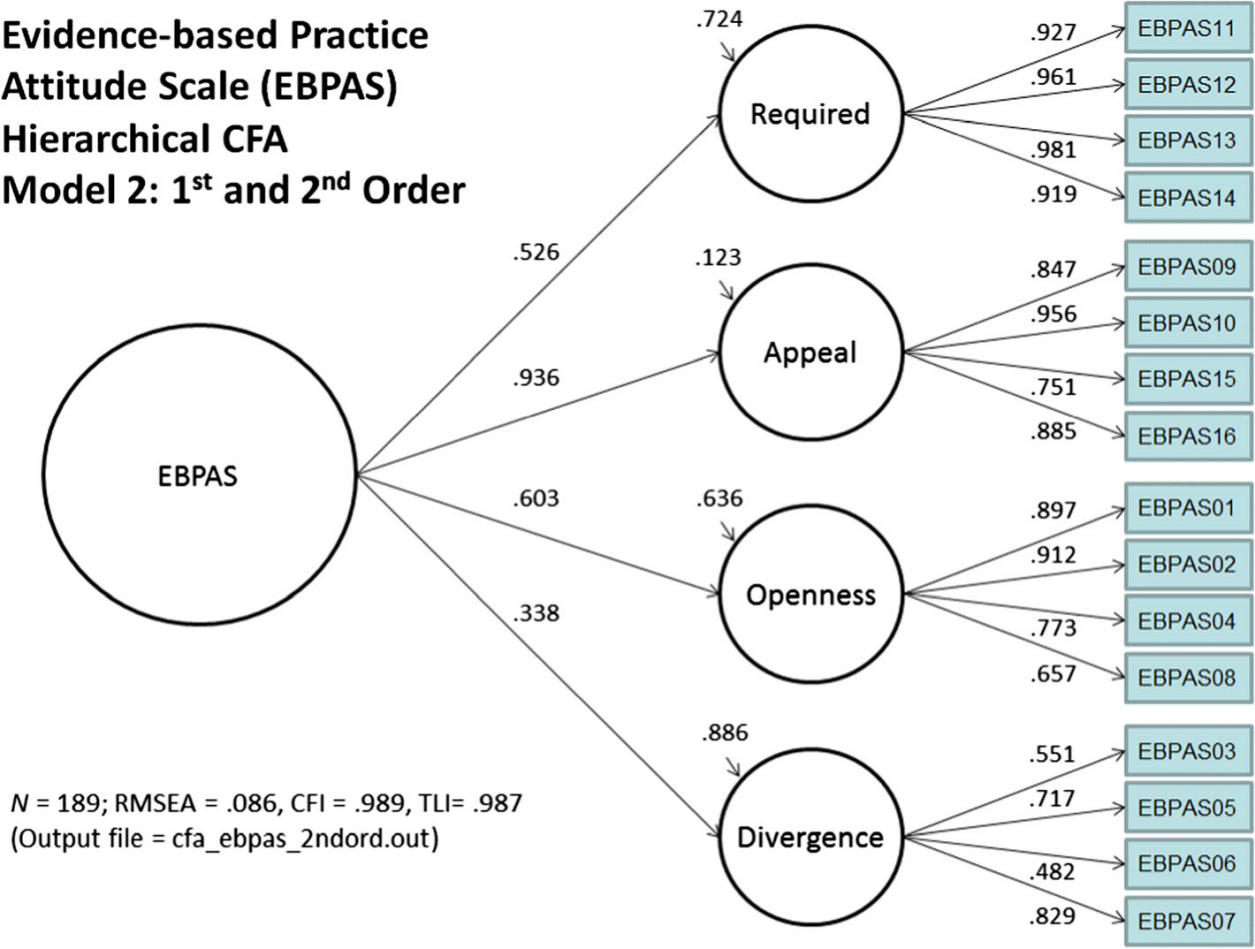
Results of confirmatory factor analysis with first- and second-order factors

The construct validity of the school-adapted EBPAS was assessed with two separate CFA models. The first model examined the four theorized sub-constructs without a higher second-order factor capturing a total attitude score. The second model was a hierarchical CFA with items loading on the four theorized first-order factors that, in turn, loaded on a second-order total score capturing overall attitudes. The second hierarchical CFA model fit the data slightly better than the first model. Results of both of the models are included as an Additional file 2, but only the structural model (Fig. 1) and results for the hierarchical CFA are reported here. Fit statistics for the second model were *χ*^2^ (df = 100, *n* = 189) = 240.13, *p* < .001, CFI = .989, TLI = .987, RMSEA = .086 (90% confidence interval = .072 to .100). All standardized item factor loadings were significant (*p* < .05; *ß*s > .480) across all the subscales. Moreover, the first-order factor loadings onto the second-order factor were all significant (*p* < .05); three of the factors had large standardized factor loadings (*ß*s > .525; Requirements, Appeal, and Openness) and one had a moderate factor loading (*ß*s > .338; Divergence). Separate CFAs were performed for each of the subscales to examine whether the overall model masked poor fit of the individual subscales. Results from these models indicated adequate fit for each of the subscales (e.g., CFI > .984, TFI > .952) and all factor loadings significant and above *ß*s > .480.

A correlation matrix depicting the associations between the EBPAS total score and four first-order factors are shown in Table 3. All of the correlations were derived from interfactor correlations between the subscales, and the total score was significant. The strongest correlations were noted between the EBPAS total score and four subscales. Of the subscales, Appeal had the strongest correlations with the other subscales, while Divergence had the weakest.

**Table 3:** Interfactor correlations across EBPAS scores

	EBPAS: Total	EBPAS: Requirements	EBPAS: Appeal	EBPAS: Openness	EBPAS: Divergence
EBPAS: Total	1.0	–	–	–	–
EBPAS: Requirements	0.52**	1.0	–	–	–
EBPAS: Appeal	0.94**	0.51^**^	1.0	–	–
EBPAS: Openness	0.60**	0.28^*^	0.56^**^	1.0	–
EBPAS: Divergence	0.34**	0.15*	0.21*	0.40^**^	1.0

**Correlation is significant at the 0.01 level (two-tailed)

*Correlation is significant at the 0.05 level (two-tailed)

### Criterion-related validity

Bivariate correlational analyses between the EBPAS scores and three consultation-related variables were performed to examine evidence of criterion-related validity (see Table 4). The EBPAS total score has significant, positive correlations with two of the three criterion variables: consultant use of behavior techniques and embeddedness in a given school. Results at the subscale level indicated that the Openness subscale was significantly and positively associated with all three criterion variables, with the strongest association found for reported use of implementation strategies. Openness also was the only EBPAS subscale to significantly predict consultant self-efficacy, indicating that those who were more open to EBP also had higher self-efficacy. The only other subscale found to significantly correlate with the criterion- related variables was Requirements, which had positive correlations with two of the three variables: use of implementation strategies and consultant embeddedness.

**Table 4:** Correlations between EBPAS subscales and consultation variables

	EBPAS total: Overall Attitudes	EBPAS: Requirements	EBPAS: Appeal	EBPAS: Openness	EBPAS: Divergence
Consultant self-efficacy	0.10	0.09	0.01	0.20^*^	0.08
Number of strategies used	0.23^**^	0.16^*^	0.12	0.29^**^	0.04
Consultant embeddedness	0.23^**^	0.27^**^	0.06	0.21^**^	0.01

**Correlation is significant at the 0.01 level (two-tailed)

*Correlation is significant at the 0.05 level (two-tailed)

## Discussion

The purpose of this study was to adapt and confirm the underlying factor structure and technical adequacy of the EBPAS when administered in the educational sector to school-based behavioral health consultants. Findings from the confirmatory analyses were consistent with the factor structure and psychometric properties found in the original study in a sample of public sector mental health providers [6]. Results supported a model with four first-order factors (Openness, Requirements, Appeal, and Divergence) loading onto a higher order factor reflecting general evidence-based practice attitudes. Coefficient alphas demonstrated strong internal consistency, with the exception of the Divergence subscale which, consistent with the original EBPAS validation study [6], fell slightly below the conventional acceptable level (*α* < .70) [34]. Correlations among the subscales indicated both unique and shared variance, with Appeal demonstrating the strongest correlations with all other subscales and the total score. It is possible that an attitudinal category like Appeal may serve to influence other types of attitudes (e.g., openness to adopt and implement), because providers or intermediaries for whom EBPs have no appeal are unlikely to be open to adopting and implementing EBPs. Lastly, correlations between EBPAS scores and consultant-relevant variables provided evidence supporting differential criterion-related validity across subscales, with small to moderate correlations revealed for only the total score and Openness and Requirements subscales. The following section discusses the implications of the findings for future research examining attitudes toward EBP.

### Implications for efforts to measure and address evidence-based practice attitudes

The adapted version of the EBPAS performed well when administered to behavioral health consultants operating in schools, supporting the relevance of assessing attitudes in school settings. In general, educational professionals who function in consultative roles tend to endorse more supportive beliefs regarding the incorporation of EBPs into routine school-based service delivery than teachers [35, 36]. Moreover, on average, the mean scores obtained from this study’s sample were higher across all subscales when compared to previous studies using the EBPAS with providers [6, 8, 37, 38]. Unlike consultants, frontline providers who are responsible for the delivery of an EBP may have different attitudes about taking on new practices because adoption requires them to change their professional routines and behavior, potentially resulting in (a) less EBP appeal; (b) less openness; and (c) more negative reactions to EBP requirements than personnel in consultative roles. Future research should explore attitude alignment among different professional roles (e.g., providers, administrators/supervisors, intermediaries) and whether discrepancies predict implementation outcomes. Moreover, attitudes reflecting appeal are most strongly related to the EBPAS total score,

In light of the confirmatory evidence, the EBPAS could be applied in the education sector as a measure to examine the impact of efforts to alter educational professionals’ attitudes with the goal of creating greater commitment to undertake EBP implementation among providers and consultants. If employed at the beginning of an EBP adoption process, the EBPAS could help inform efforts to prepare a setting organization for initial implementation, as favorable attitudes among professionals is a component of organizational readiness for change [39, 40]. Implementation strategies informed by the attitude change literature could be particularly helpful to promote more favorable attitudes among implementation practitioners [41]. There are efforts underway in the educational sector to develop and test pre-implementation strategies targeting providers’ attitudes among other putative mechanisms of behavior change [42, 35]; however, there are no known efforts targeting attitudes among consultants or other personnel supporting EBP implementation.

The correlational analyses suggested that attitudes were associated with consultant embeddedness (i.e., visibility and connections to others in the service setting) and use of implementation strategies. These findings suggest that attitudes may be associated with consultant behavior, which, in turn, has the potential to impact providers’ EBP implementation [43]. Most consultation models assume that consultants have favorable attitudes. This may not be the case universally, as indicated by the variability among the respondents in this study. If consultant attitudes are unfavorable, they may be less likely to put in the effort required to influence implementation outcomes (e.g., collaborating on EBP implementation and using implementation strategies).

### Limitations/directions for future research

Further study will be needed to examine the temporal reliability of the EBPAS and provide a more extensive assessment of validity, as this study examined only internal consistency and a limited set of potential criterion-related variables. Given the variability among educational systems across the globe [44], the generalizability of the current findings and school-adapted EBPAS beyond US schools is unclear and should be examined. Furthermore, this study did not link EBPAS scores to actual implementation outcomes (such as adoption, fidelity, and reach), or the subsequent behavioral health outcomes. Behavioral health consultants tend to sit at the center of school-based behavioral health implementation efforts yet reflect only one role in a school among other professionals who might be involved in implementation efforts [16, 45]. Data gathered from multiple informants and across multiple roles are likely to yield important insights into the importance of attitudes for EBP implementation effectiveness.

## Conclusions

This study expanded extant EBPAS research by adapting and validating the instrument for use in the educational sector with behavioral health consultants. This research extends the external validity of the EBPAS not only to a novel service setting (i.e., schools), but a different group of stakeholders involved in the implementation process (i.e., consultants). Despite this study’s confirmatory findings, there remain several avenues for future research that explore applications and adaptations to the measure. Differential criterion-related validity estimates bring into question the Divergence subscale, which may not serve as a valid sub-construct of attitudes when used with consultants. Moreover, research that examines the application of EBPAS to inform and evaluate the impact of implementation strategies that target professionals’ attitudes as a key mechanism of implementation outcomes should be prioritized.

## Additional file

Additional file 1: Consultative-relevant Implementation Strategies. (DOCX 13 kb)

Additional file 2: Supplemental File_CFA models. (DOCX 118 kb)

## Abbreviations

CFA: Confirmatory factor analysis
CFI: Comparative fit index
DV: Dependent variable
EBP: Evidence-based practice
EBPAS: Evidence-Based Practice Attitude Scale
RMSEA: Root mean square error of approximation
TLI: Tucker-Lewis index
WLSMV: Weighted least squares means and variances
