# Supplementary material for: Confirmatory factor analysis of the Evidence-Based Practice Attitudes Scale with school-based behavioral health consultants
Source: Implement Sci. 2018 Aug 22;13:116. doi: 10.1186/s13012-018-0804-z (PMC6106841; doi:10.1186/s13012-018-0804-z)
Supplement: Supplementary file 1 — Consultative-relevant Implementation Strategies. (DOCX 13 kb) [file 13012_2018_804_MOESM1_ESM.docx]

**Additional File 1**

**Consultative-relevant Implementation Strategies**

1. Providing performance based feedback
2. Providing incentives (e.g., offer to cover class, relief of a duty, gift cards, letters of recommendation, etc.) to implement the intervention
3. Modeling the implementation of specific features of the intervention
4. Distributing educational materials to support implementation (e.g., providing an intervention script that outlines precisely how to implement it with fidelity)
5. Providing the implementer with choices about which intervention or how to implement the intervention
6. Showing research evidence that demonstrates how the intervention works
7. Using testimonials from others who have experienced success with the intervention
8. Pre-correcting problems by checking in with the implementer immediately before implementation begins and reminding him/her what successful implementation entails
9. Giving rationales for how the intervention will make life better for the implementer
10. Creating a learning collaborative (e.g., professional learning community)
11. Providing ongoing training (i.e., introducing new content over time)
12. Providing ongoing consultation (for content already covered in training)
13. Shadow other experts (i.e., directly observe experienced people use the intervention)
14. Use train-the-trainer strategies (i.e., train individuals who will then train others)
15. Make training dynamic (i.e., use interactive method and vary the information delivery methods to cater to different learning styles and work contexts)
