# Supplementary figures and images for: Confirmatory factor analysis of the Evidence-Based Practice Attitudes Scale with school-based behavioral health consultants
Source: Implement Sci. 2018 Aug 22;13:116. doi: 10.1186/s13012-018-0804-z (PMC6106841; doi:10.1186/s13012-018-0804-z)

Supplemental File


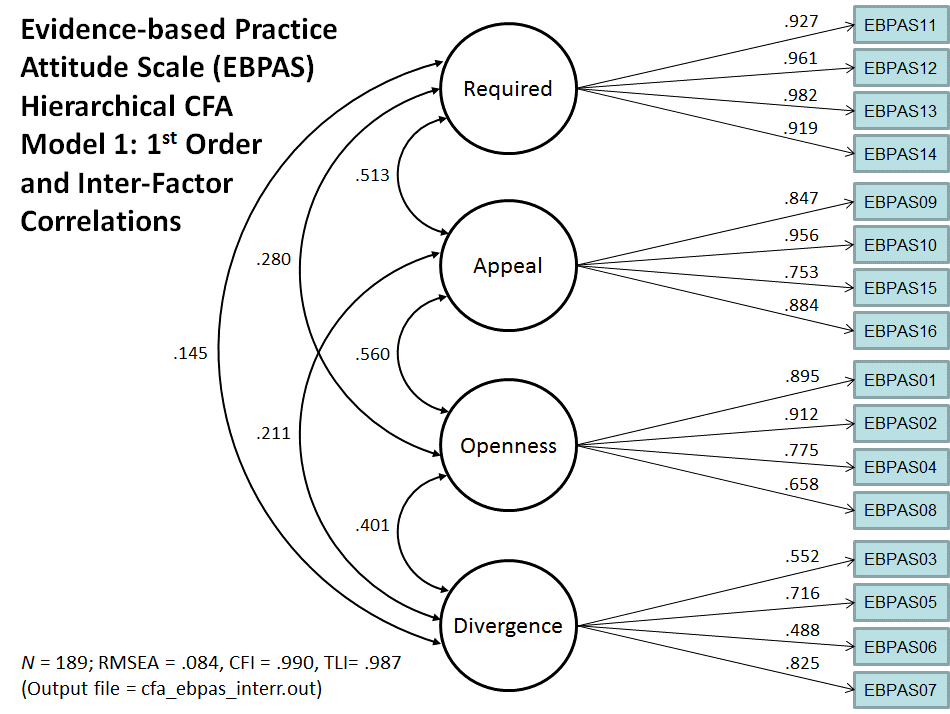


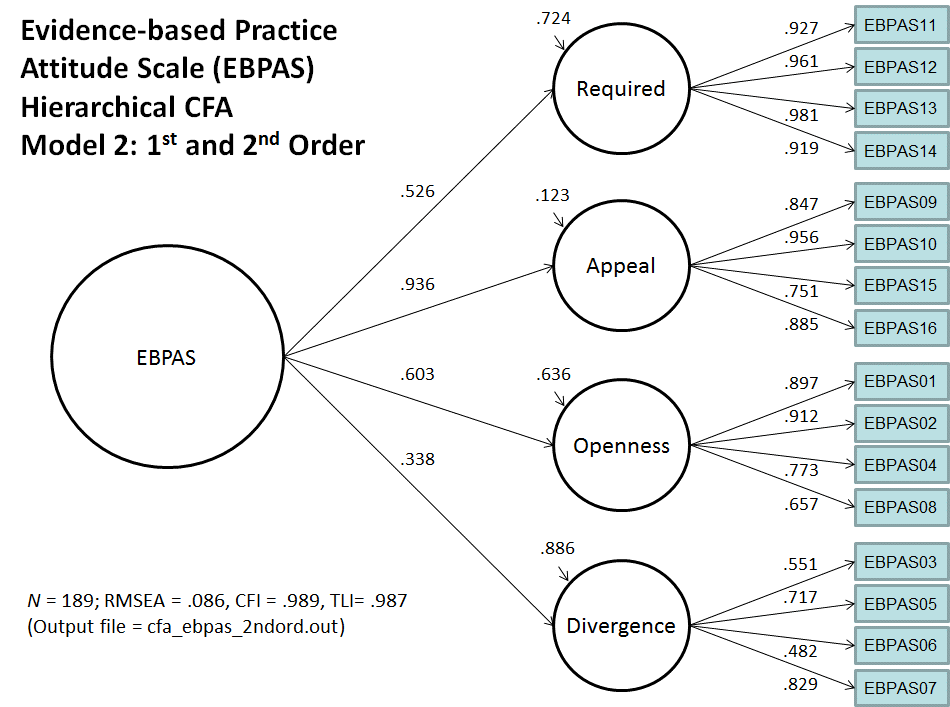

Supplement: Supplementary file 2 — Supplemental File_CFA models. (DOCX 118 kb) [file 13012_2018_804_MOESM2_ESM.docx]
